# Correction: Kummerowia striata extract protects paracetamol-induced liver injury by modulating the S1P/Nrf2/Keap1 pathway

**DOI:** 10.1371/journal.pone.0356628

**Published:** 2026-08-18

**Authors:** Huanghui Qin, Runxiao Chen, Youlan Xie, Hang Liu, Yubo Xiao, Lanyu Li

The following information is missing from the Funding statement: The Project for Enhancing the Basic Research Capacity of Young and Middle-aged Teachers in Guangxi Universities (Grant No. 2021KY0518 to Lanyu Li).
