# Supplementary figures and images for: Kummerowia striata extract protects paracetamol-induced liver injury by modulating the S1P/Nrf2/Keap1 pathway
Source: PLoS One. 2025 Aug 14;20(8):e0329837. doi: 10.1371/journal.pone.0329837 (PMC12352777; doi:10.1371/journal.pone.0329837)

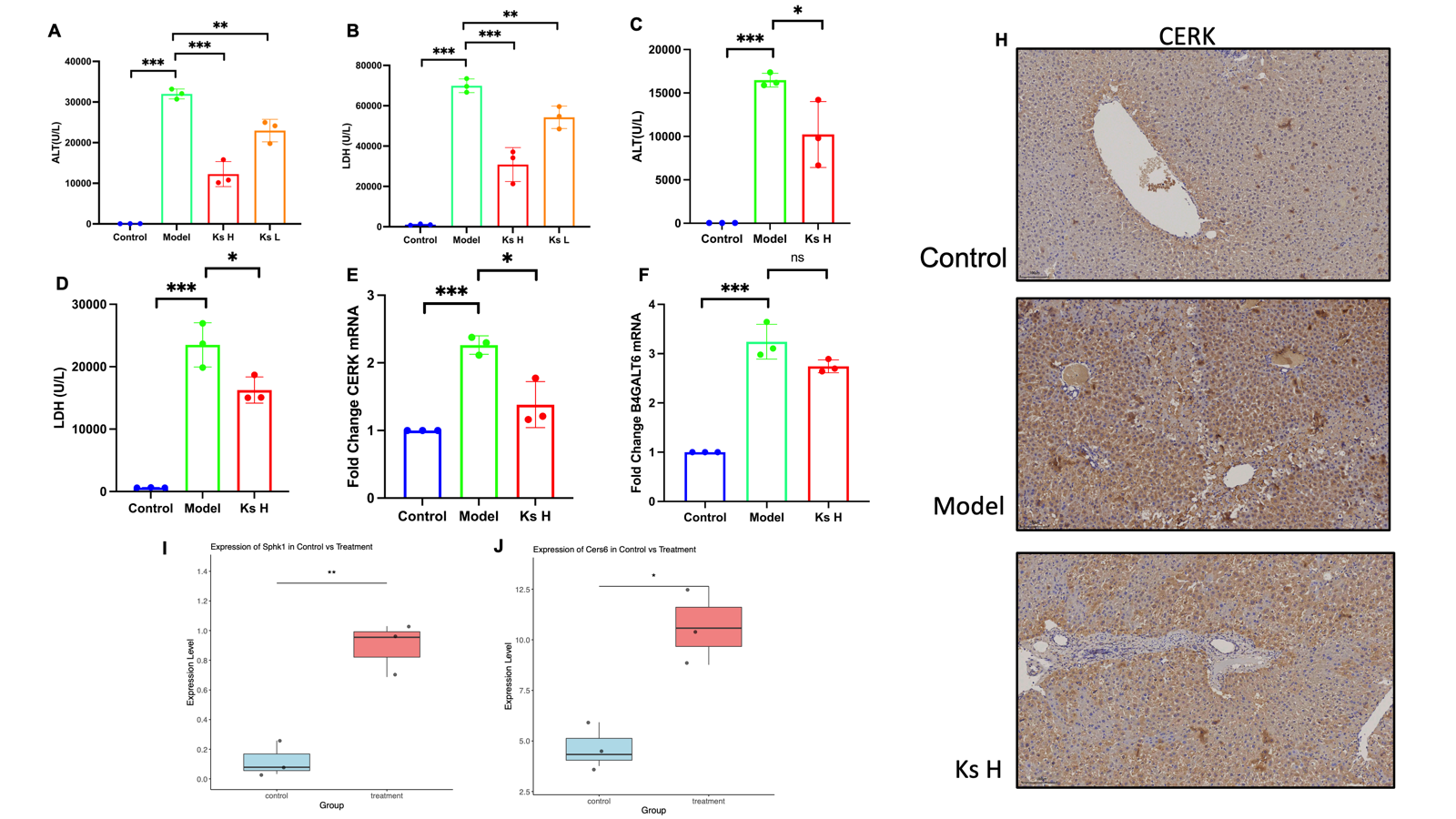

Supplement: S1 Fig — (C-D) Changes in serum ALT and LDH levels in female Km mice with acute liver injury induced by CCl₄ following Ks treatment.(E, H) mRNA expression of CERK in liver tissues of male C57/B6J mice with acute liver injury induced by APAP, and IHC staining images.(F) mRNA expression of B4GALT6 in liver tissues of male C57/B6J mice with acute liver injury induced by APAP following Ks treatment.(I,J)Waterfall plot of the most significantly altered sphingolipid metabolic pathway genes (SPHK1, Cer S6) in dataset GSE241511. (JPG) [file pone.0329837.s001.jpg]

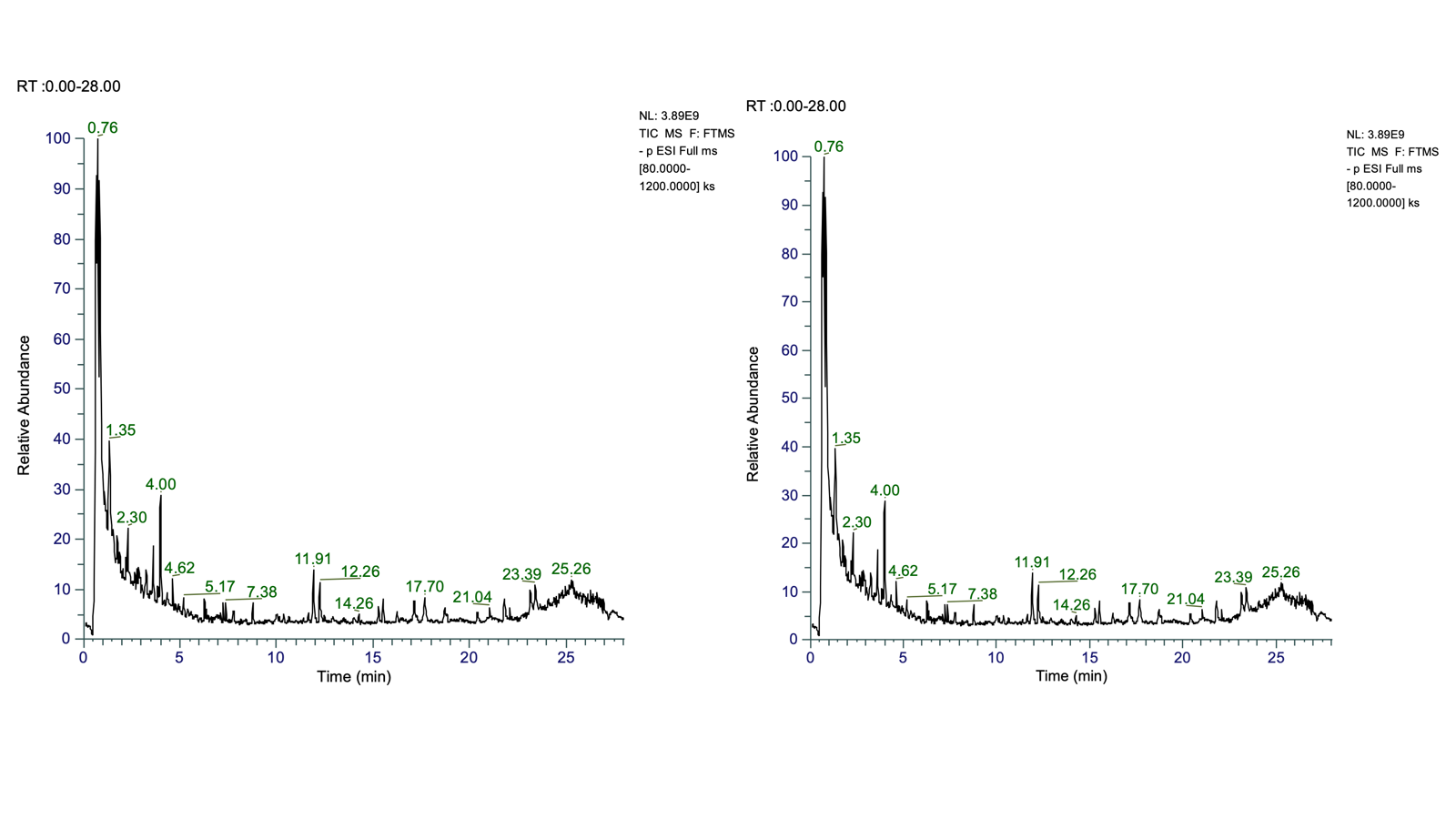

Supplement: S3 Fig — (JPG) [file pone.0329837.s003.jpg]

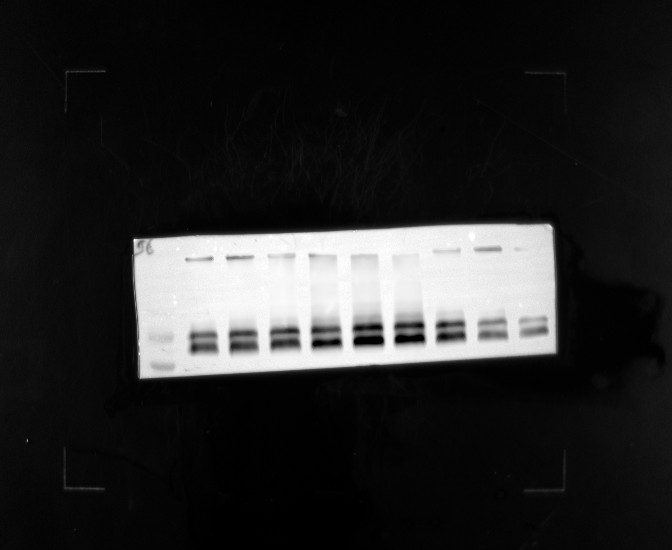

Supplement: S1 Raw_images — (ZIP) [file pone.0329837.s004.zip › western blot/Fig3 Keap1 68kda-shine-merger.tif]

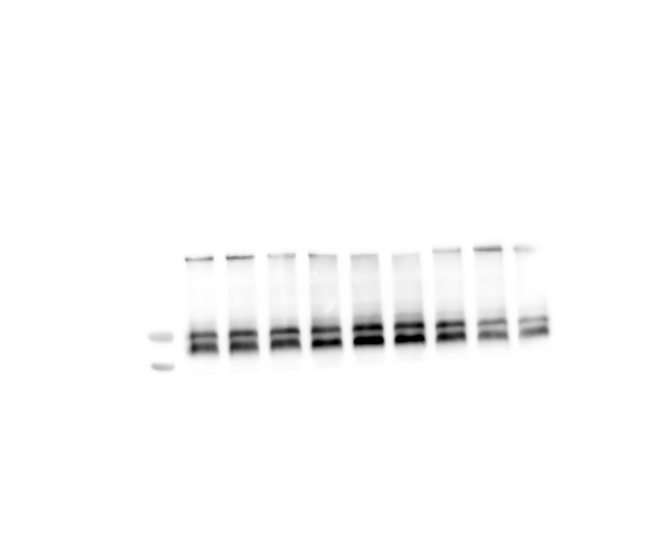

Supplement: S1 Raw_images — (ZIP) [file pone.0329837.s004.zip › western blot/Fig3 Keap1 68kda.tif]

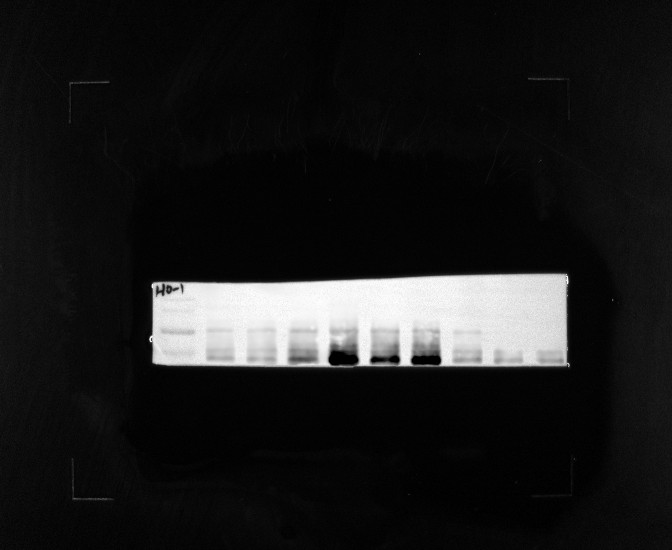

Supplement: S1 Raw_images — (ZIP) [file pone.0329837.s004.zip › western blot/Fig3 Nrf2 110kda-shine-merger.tif]

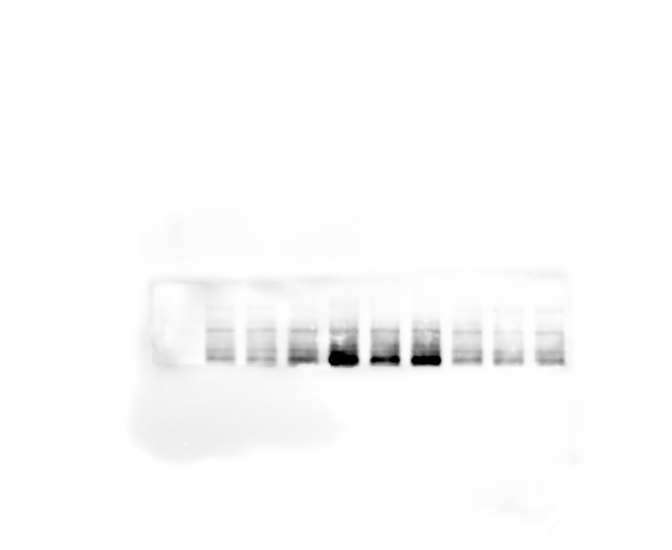

Supplement: S1 Raw_images — (ZIP) [file pone.0329837.s004.zip › western blot/Fig3 Nrf2 110kda-shine.tif]

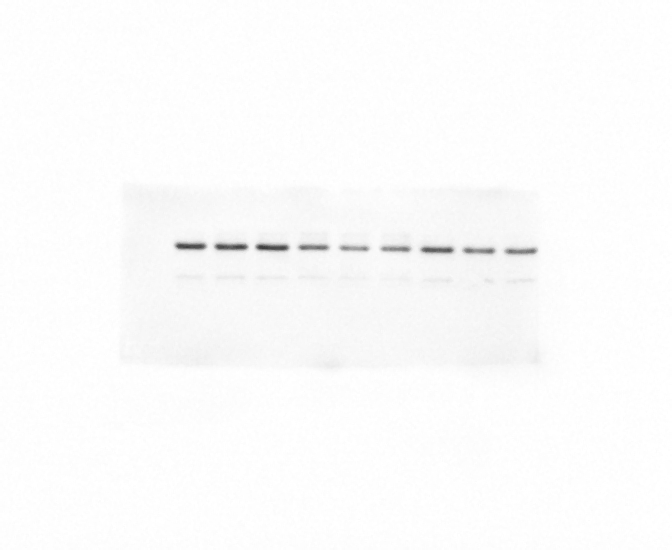

Supplement: S1 Raw_images — (ZIP) [file pone.0329837.s004.zip › western blot/Fig3 Nrf2 _GAPDH 36kda-shine.tif]

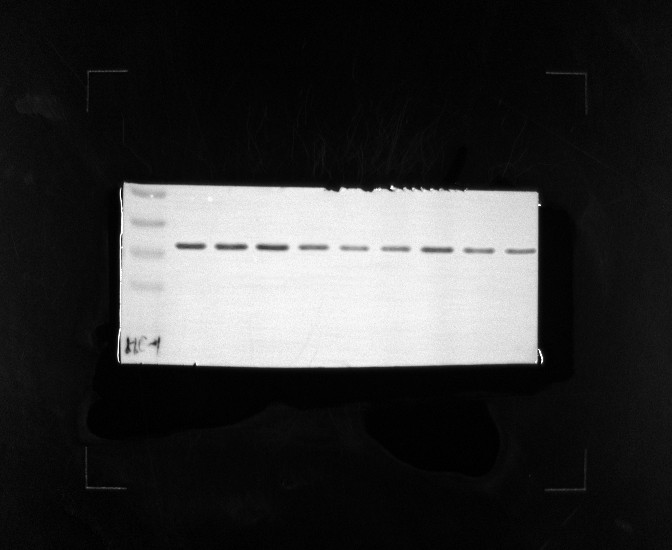

Supplement: S1 Raw_images — (ZIP) [file pone.0329837.s004.zip › western blot/Fig3 Nrf2 _GAPDH-36kda-shine-merger.tif]

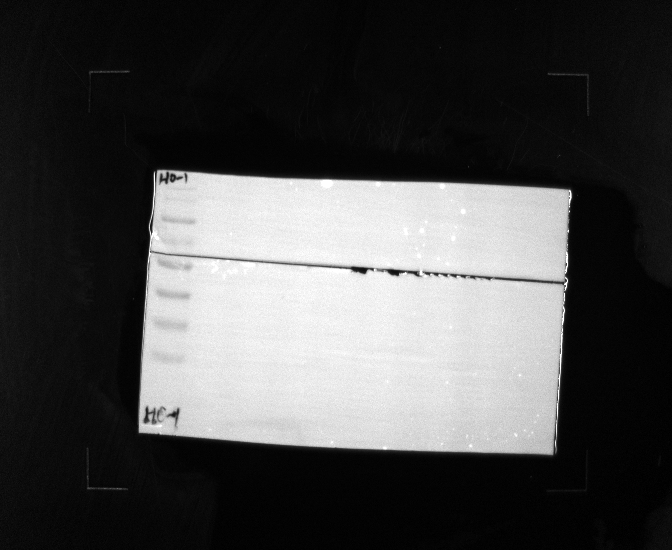

Supplement: S1 Raw_images — (ZIP) [file pone.0329837.s004.zip › western blot/Fig3 Nrf2 and GAPDH splicing full film-bright.tif]

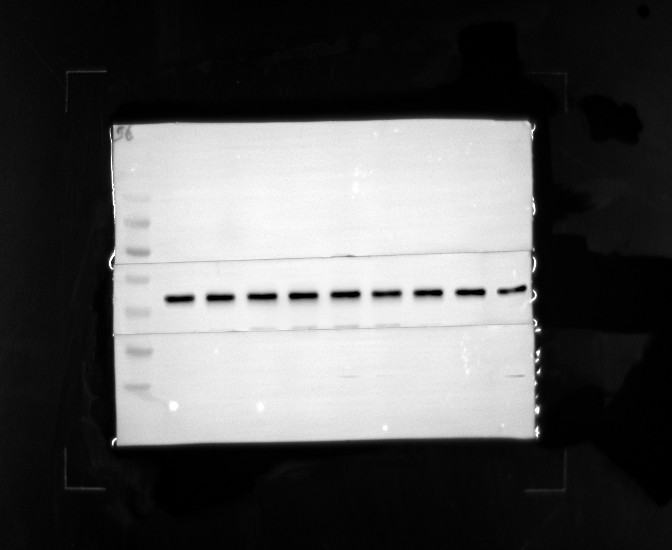

Supplement: S1 Raw_images — (ZIP) [file pone.0329837.s004.zip › western blot/Fig3 and 4-Keap1+SPHK1_GAPDH 36kda-shine-merger.tif]

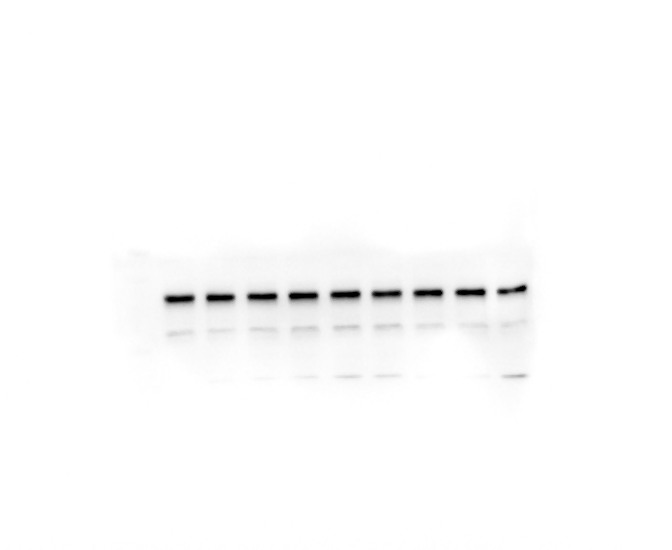

Supplement: S1 Raw_images — (ZIP) [file pone.0329837.s004.zip › western blot/Fig3 and 4-Keap1+SPHK1_GAPDH 36kda-shine.tif]

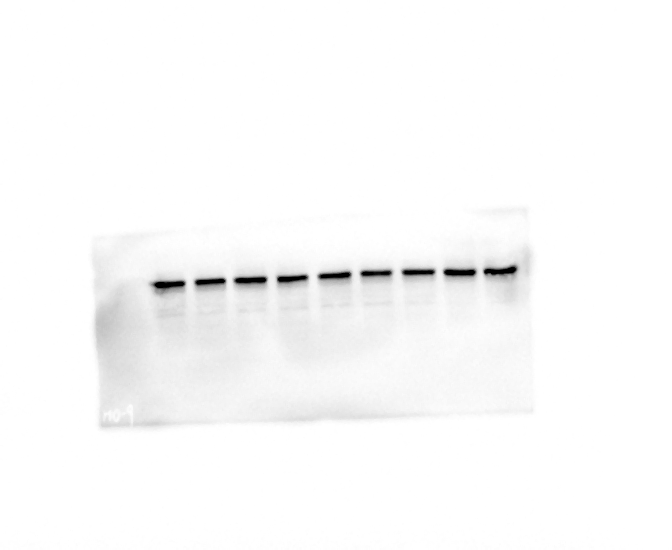

Supplement: S1 Raw_images — (ZIP) [file pone.0329837.s004.zip › western blot/Fig4 HO-1 -GAPDH 36kda-shine.tif]

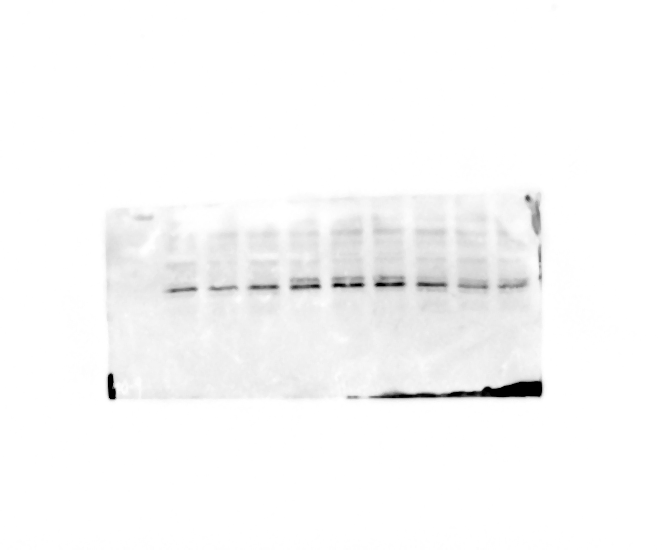

Supplement: S1 Raw_images — (ZIP) [file pone.0329837.s004.zip › western blot/Fig4 HO-1 31kda-shine.tif]

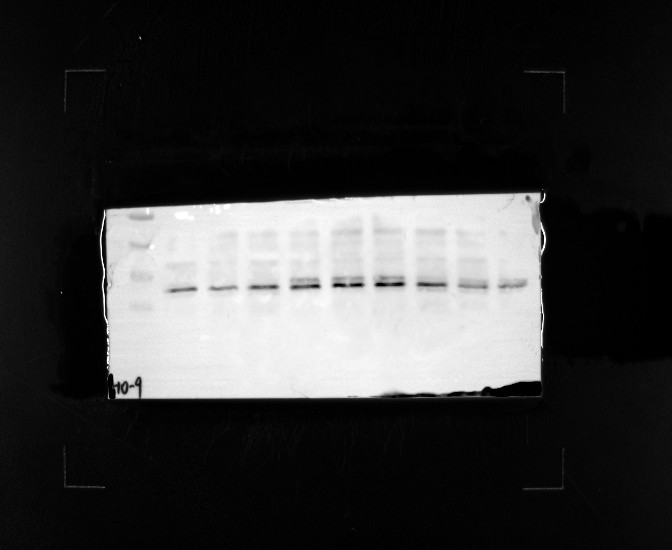

Supplement: S1 Raw_images — (ZIP) [file pone.0329837.s004.zip › western blot/Fig4 HO-1 31kda.tif]

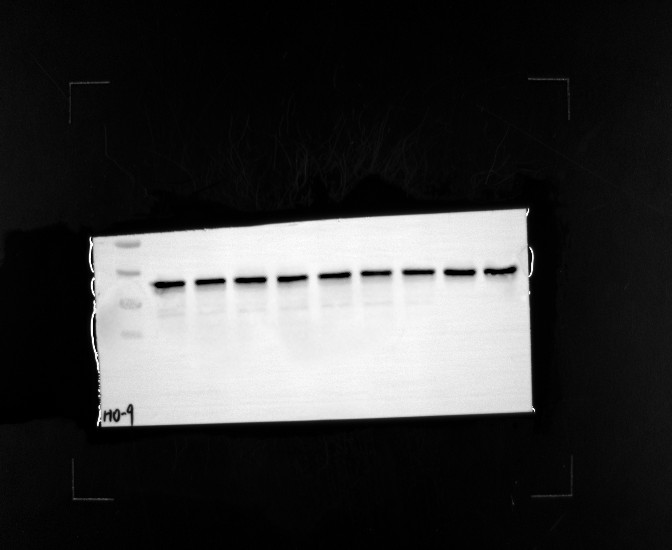

Supplement: S1 Raw_images — (ZIP) [file pone.0329837.s004.zip › western blot/Fig4 HO-1 _GAPDH 36kda-shine-merger.tif]

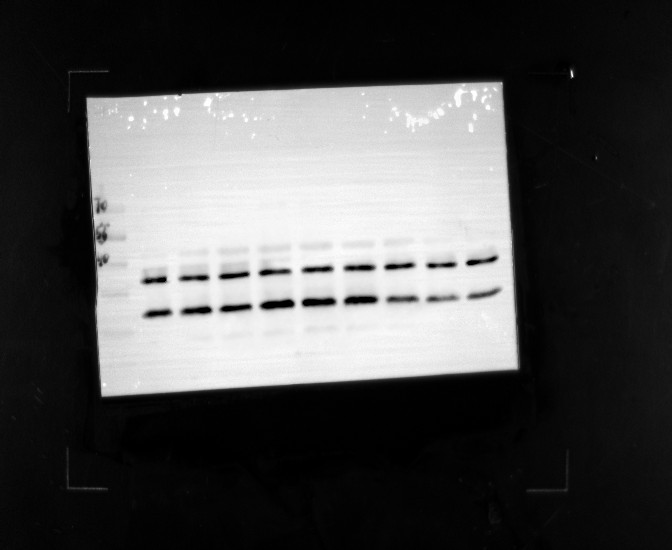

Supplement: S1 Raw_images — (ZIP) [file pone.0329837.s004.zip › western blot/Fig4 NQO1 33kda+GAPDH 36kda-shine-merger.tif]

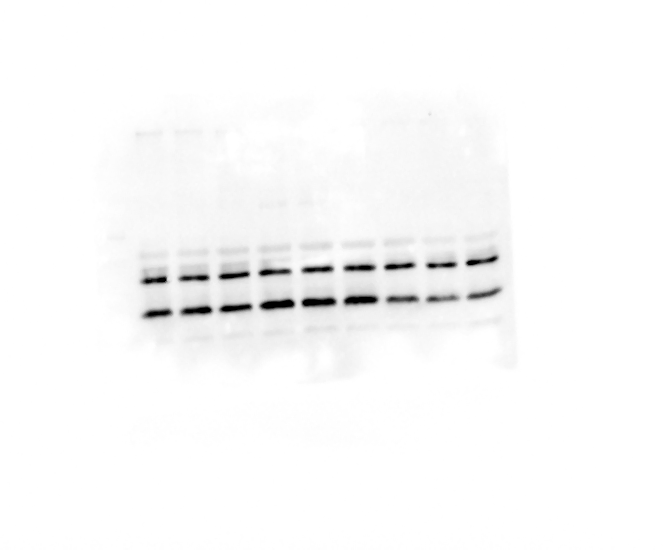

Supplement: S1 Raw_images — (ZIP) [file pone.0329837.s004.zip › western blot/Fig4 NQO1 33kda+GAPDH 36kda-shine.tif]

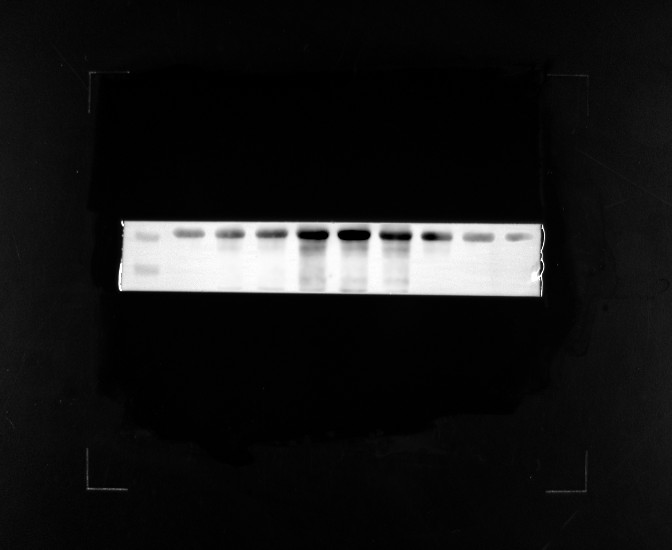

Supplement: S1 Raw_images — (ZIP) [file pone.0329837.s004.zip › western blot/Fig4 SPHK1 42kda-shine-merger.tif]

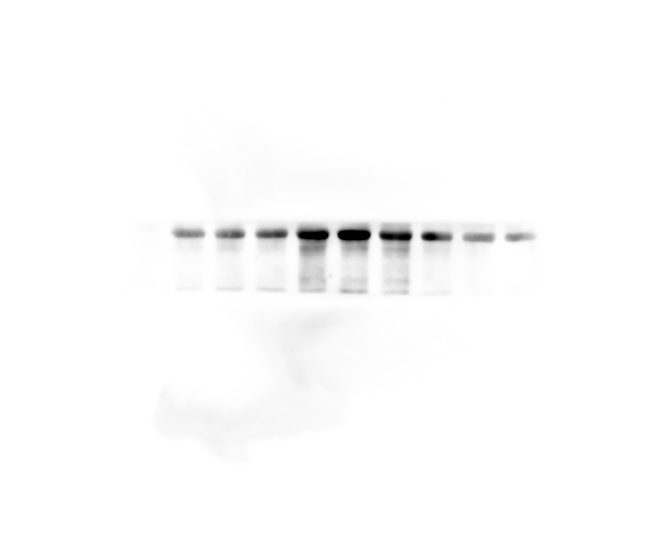

Supplement: S1 Raw_images — (ZIP) [file pone.0329837.s004.zip › western blot/Fig4 SPHK1 42kda-shine.tif]
